# Supplementary material for: Poor Diet, Long Sleep, and Lack of Physical Activity Are Associated with Inflammation among Non-Demented Community-Dwelling Elderly
Source: Healthcare (Basel). 2022 Jan 12;10(1):143. doi: 10.3390/healthcare10010143 (PMC8775386; doi:10.3390/healthcare10010143)
Supplement: Supplementary file 1 [file healthcare-10-00143-s001.zip › healthcare-1481802-supplementary.pdf]

Supplementary Material

Supplementary Table S1. Zero order and partial correlations of variables of interest with levels of IL-6. † $p < 0.01$.

**Confounders**	**–**	**Age, Gender, BMI**	**Age, Gender, BMI, MCI Diagnosis**		**–**	**Age, Gender, BMI**	**Age, Gender, BMI, MCI Diagnosis**
Night TST	0.03	0.01	0.01	Mediterranean Diet Score	0.03	0.01	0.01
24–h TST	0.04	0.03	0.02	Consumption of:			
Night TMB	0.07	0.04	0.03	Vegetables	−0.28 †	−0.33 †	−0.36 †
24–h TMB	0.08	0.08	0.06	Red meat	−0.11	−0.12	−0.12
Night Sleep Efficiency	−0.04	−0.04	−0.02	Dairy poducts	−0.07	−0.11	−0.11
Night WASO	0.03	0.02	0.01	Legumes	−0.12	−0.12	−0.13
Night Sleep latency	0.13	0.13	0.13	Non−refined cereal	−0.11	−0.06	−0.05
Number of Awakenings	−0.02	0.01	0.01	Refined cereal	−0.02	−0.04	−0.04
Sleep duration	0.09	0.08	0.08	Potatoes	−0.14	−0.15	−0.15
Non−refreshing sleep	0.04	0.04	0.05	Fruit	−0.02	−0.03	−0.02
Leg movement	−0.09	−0.12	−0.13	Fish	−0.02	−0.04	−0.04
EDS	−0.06	−0.06	−0.07	Poultry	0.01	−0.02	−0.03
Sleep Apnea symptoms	−0.04	−0.01	0.01	Eggs	−0.11	−0.12	−0.12
Insomnia symptoms	−0.06	−0.05	−0.09	Sweets	−0.09	−0.14	−0.14
Physical activity	−0.29 †	−0.29 †	−0.29 †	Alcoholic beverages	−0.02	−0.13	−0.12

Total Energy Intake	−0.06	−0.11		−0.10

Supplementary Table S2. Zero order and partial correlations of variables of interest with levels of TNF-a. * $p < 0.05$.

Confounders	–	Age, Gender, BMI	Age, Gender, BMI, MCI Diagnosis		–	Age, Gender, BMI	Age, Gender, BMI, MCI Diagnosis
Night TST	0.07	0.03	0.02	Mediterranean Diet Score	−0.05	−0.10	−0.08
24−h TST	0.04	0.02	0.01	Consumption of:			
Night TMB	0.10	0.04	0.03	Vegetables	−0.21 *	−0.19 *	−0.18 *
24−h TMB	0.04	0.02	0.02	Red meat	−0.17	−0.12	−0.14
Night Sleep Efficiency	0.001	0.02	0.03	Dairy products	0.09	0.13	0.13
Night WASO	0.05	0.02	0.01	Legumes	−0.03	−0.03	−0.04
Night Sleep latency	0.09	0.07	0.06	Non−refined cereal	0.05	0.10	0.13
Number of Awakenings	0.06	0.09	0.09	Refined cereal	−0.01	−0.02	−0.01
Sleep duration	0.06	0.06	0.08	Potatoes	−0.13	−0.14	−0.14
Non−refreshing sleep	0.002	−0.06	−0.07	Fruit	0.01	0.01	0.01
Leg movement	−0.13	−0.14	−0.15	Fish	−0.10	−0.09	−0.09
EDS	−0.02	−0.04	−0.05	Poultry	0.01	0.07	0.06
Sleep Apnea symptoms	−0.14	−0.09	−0.10	Eggs	0.11	0.09	0.13
Insomnia symptoms	−0.11	−0.13	−0.13	Sweets	−0.16	−0.13	−0.11
Physical activity	−0.05	−0.04	−0.05	Alcoholic beverages	0.10	0.07	0.09
Total Energy Intake	−0.04	−0.01	−0.04				
